# Functional outcomes and complications of distal humerus hemiarthroplasty performed in acute versus salvage cases: a systematic meta-analysis

**DOI:** 10.1016/j.jseint.2026.101639

**Published:** 2026-01-27

**Authors:** Cerise Gosselin, Kevin A. Hao, Nicolas Bonnevialle, Pierre Mansat, Hugo Barret

**Affiliations:** a Chirurgie Orthopédique et Traumatologique, Centre Hospitalier Universitaire de Purpan Toulouse, Toulouse, France; b Department of Orthopaedic Surgery & Sports Medicine, University of Florida, Gainesville, FL, USA

**Keywords:** Distal humerus, Comminuted, Hemiarthroplasty, Elbow fracture, Acute, Salvage, ORIF failure, Meta-analysis

## Abstract

**Background:**

Distal humeral hemiarthroplasty (DHH) is a treatment option for comminuted joint fractures of the elbow, particularly when osteosynthesis is not possible or has failed. This meta-analysis aims to compare the clinical and functional outcomes of DHH performed for distal humerus fractures acutely versus as salvage after failure of osteosynthesis (open reduction and internal fixation [ORIF]).

**Methods:**

A systematic review was conducted according to Preferred Reporting Items for Systematic reviews and Meta-Analyses guidelines. Studies reporting cases of DHH for complex distal humerus fractures were included and grouped according to the surgical context: acute (initial fracture) or salvage (ORIF failure). The variables analyzed included functional scores (Mayo Elbow Performance Score, Disabilities of the Arm, Shoulder and Hand), joint range of motion (flexion, extension, pronation-supination), complication rates, reoperation rates, and revision rates. Comparisons were performed using random-effects meta-analysis.

**Results:**

Eighteen studies totaling 583 patients (547 acute; 36 salvage) met inclusion. Functional scores were comparable between groups (Mayo Elbow Performance Score, 86 vs. 83; Disabilities of the Arm, Shoulder and Hand, 16.7 vs. 17.3). Pooled complication rates were 26.5% in acute cases and 19.3% in salvage cases; between-group differences were not statistically significant in our meta-analytic comparison.

**Conclusion:**

DHH offers comparable functional outcomes and similar complication rates whether performed acutely or as salvage after failed ORIF. Prospective and adequately powered comparative studies are needed to refine indications and quantify risks.

Distal humeral fractures are a therapeutic challenge, particularly in elderly patients or those with complex comminuted fractures. Locked plate osteosynthesis is generally considered the treatment of choice when feasible.^7^ However, in cases of osteoporotic bone, severe joint comminution or failure to heal, arthroplasty is a viable treatment option. Total elbow arthroplasty (TEA) was a well-established alternative for elderly patients with low functional demands. However, it has significant drawbacks, including lifelong weight-bearing restrictions and the risk of polyethylene wear, component loosening, and long-term mechanical complications.^7^^,^^17^ In active patients, TEA is not a suitable surgical option.

Distal humeral hemiarthroplasty (DHH) has emerged as a new treatment option for complex distal humerus fractures, avoiding constrained implants (TEA) and allowing faster functional recovery without weight-bearing restrictions.^19^^,^^20^^,^^22^ This technique can be performed both in acute cases, for irreparable fractures, and as a salvage procedure after complicated osteosynthesis failure (eg, nonunion, hardware failure, stiffness).^8^^,^^10^ However, the reported results remain heterogeneous, and there are few comparative studies evaluating outcomes of DHH for acute versus salvage cases. Arthroplasty performed as revision or salvage is technically demanding because of scar tissue, bone stock, and soft-tissue constraints, which may influence complications and rehabilitation.^6^^,^^23^ In parallel, TEA literature suggests similar outcomes in primary vs. salvage settings after distal humerus fracture, providing useful context for DHH timing decisions.^10^^,^^17^

To date, no meta-analysis has specifically compared the functional outcomes, joint range of motion, and complication rates of DHH performed in acute versus salvage cases of distal humerus fractures. The aim of this study is therefore to compare the results of DHH performed acutely versus as a salvage procedure after failed osteosynthesis. We hypothesized that DHH performed in the acute setting would portend a superior clinical outcome compared to DHH performed as a salvage procedure.

## Methods

### Search strategy

This systematic review was conducted in accordance with the PRISMA (Preferred Reporting Items for Systematic Reviews and Meta-Analyses) 2020 guidelines.^13^ An exhaustive literature search was conducted in the PubMed/MEDLINE, Embase, and Cochrane Library databases, with no date restrictions, up to May 2025. The electronic search strategy was developed using both controlled vocabulary (MeSH/Emtree) and free-text keywords. The following terms were combined: (*“distal humerus”* OR *“elbow”*) AND (*“fracture”*) AND (*“hemiarthroplasty”* OR *“arthroplasty”*) AND (*“acute”* OR *“salvage”* OR *“revision”* OR *“conversion”* OR *“nonunion”* OR *“failed ORIF”*), applying Boolean operators AND and OR appropriately. Search syntax was adapted for each database (PubMed/MEDLINE, Embase, and Cochrane Library). A manual search of the bibliographic references of the included articles was conducted to identify other relevant studies. The review was not prospectively registered; we explicitly acknowledge this methodological limitation.

### Inclusion criteria

Studies were eligible if they met the following criteria: they were original, prospective, or retrospective clinical studies involving adult patients who had undergone DHH for comminuted joint fractures of the elbow. The studies had to include patients who underwent either acute surgery (primary surgery performed within days of the trauma) or salvage surgery (secondary surgery after failure of osteosynthesis), or both. They had to report at least 1 outcome measure of interest, such as a functional score (Mayo Elbow Performance Score [MEPS] or the Disabilities of the Arm, Shoulder and Hand [DASH] score), joint range of motion, or complication rate. Studies reporting only cases of TEA without DHH, abstracts alone, letters to the editor without original data, and narrative reviews were excluded.

### Study selection and data extraction

Two authors (C.G. and H.B.) independently selected studies based on titles and abstracts, then read the full text. Disagreements were resolved by consensus or by consulting a third reviewer (N.B.). The data extracted included study characteristics (type, year, country, level of evidence, follow-up duration, number of patients, distribution between acute and salvage groups), demographic data (mean age, sex, laterality, dominance), and clinical data (type of implant used, surgical approach). The outcome measures included functional scores (MEPS and DASH), joint range of motion (flexion, extension, pronation, supination, functional arc), and all-cause complication rates. Data from studies reporting only 1 group (acute or salvage) were classified according to the surgical context and included in an indirect comparative analysis by subgroups. Risk of bias was qualitatively assessed using the ROBINS-I tool for nonrandomized studies.^21^ Two reviewers performed independent assessments with consensus adjudication. Overall, most studies carried a moderate to serious risk of bias, mainly due to their retrospective design, heterogeneity in salvage indications, and incomplete reporting of complications and revisions.

### Statistical analysis

Demographics and clinical outcomes of interest were tabulated. Meta-analysis was performed to compare outcome scores, ROM, and complication rates using forest plots. Studies were included for meta-analysis if they reported stratum-specific (acute and salvage cohorts) data for the outcomes of interest. We anticipated substantial heterogeneity in the design and methodology of the included studies; thus, we chose to use a random-effects model for meta-analysis *a priori*.^3^ The I^2^ statistic was used to assess the heterogeneity among data. The true effect size in 95% of the population (95% prediction interval) was calculated using the variance of true effects (*T*^*2*^) and thus the standard deviation of true effects (*T*). Meta-analysis was performed using the *metafor* package.^24^ All statistical analyses were performed using R software (version 4.2.0; R Core Team, Vienna, Austria) with an α of 0.05.

### Ethical considerations

As this study is a systematic review and meta-analysis of previously published data, it did not require approval from an ethics committee. No nonanonymized individual data were used. All included studies had been previously published in peer-reviewed journals, and all data extracted were from public sources. This review was conducted in accordance with good scientific practice and PRISMA recommendations.

## Results

**Figure 1:**
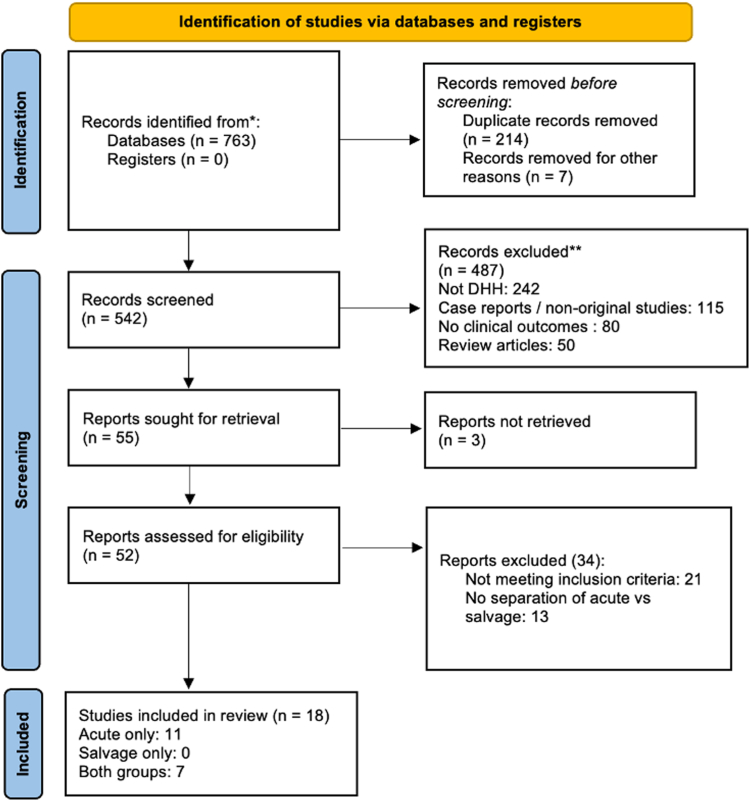
Preferred Reporting Items for Systematic reviews and Meta-Analyses (PRISMA) flow diagram showing study selection process.

Eighteen studies were included in the analysis (all retrospective), totaling 583 patients treated with DHH for comminuted distal humerus fractures (Fig. 1). Of these, 547 patients underwent acute surgery and 36 underwent salvage surgery after failure of osteosynthesis. All studies were retrospective, with sample sizes ranging from 3 to 54 patients and follow-up periods ranging from 6 to 96 months (Tables I and II).

**Table I:** Characteristics of included studies reporting outcomes of distal humeral hemiarthroplasty performed in the acute setting

Author	No. of patients	Age Mean ± SD	Follow-up Mean ± SD	MEPS Mean ± SD	DASH Mean ± SD	Flexion Mean ± SD	Extension loss Mean ± SD	FE arc Mean ± SD	Pronation Mean ± SD	Supination Mean ± SD	PS arc Mean ± SD	Complications N (%)	Revisions N (%)
Rotini et al 2023^21^	27	64 ± 7.9	82 ± 36.1	89.3	12.6 ± 8.9	121 ± 14.9	25.5 ± 15	95.5 ± 24.1	77.4 ± 8.6	75.9 ± 8.6	153.3 ± 11.9	15 (55.6%)	NR
Taylor et al 2023	293	68.5	56.4 ± 44.4									NR	20 (6.8%)
Jenkins et al 2022^11^	37	75	61		14.9	132	19	108	74	81	155	7 (18.9%)	4 (10.8%)
Schultzel et al 2022^22^	10	71.9 ± 8.8	115.2	88 ± 10.7	37.1 ± 18.4	126 ± 32.8	36 ± 17.5	84 ± 33.9	64 ± 15.4	66 ± 8.4	130 ± 19.1	3 (30%)	1 (10%)
Taylor et al 2021	8	72.1 ± 9.1	29.9	88.3 ± 5.8		135 ± 9	21 ± 15	114	87 ± 5	84 ± 8	171	2 (25%)	1 (12.5%)
Nestorson et al 2015^17^	42	72 ± 7.8	34.3 ± 8.7	90 ± 14.4	20 ± 18.1	126.8 ± 12.7	23.5 ± 16.9	103 ± 27.4	77.4 ± 12.5	77.4 ± 12.7	154.8 ± 24.8	10 (23.8%)	6 (14.3%)
Adolfsson et Nestorson 2012^2^	8	79 ± 5.8	54 ± 16.5	90.6 ± 4.9		126 ± 6.5	31 ± 11.6	96.3 ± 17.3	80	80		1 (12.5%)	0 (0%)
Heijink et al 2015^9^	1	76	21	95		115	20	95	70	80	150	0 (0%)	0 (0%)
Phadnis et al 2015^19^	16	78.7 ± 8.1	35 ± 15	89.6 ± 5	11.2 ± 5.8	130.6 ± 12.2	15 ± 9	116 ± 14	85 ± 5	85 ± 4	172 ± 9	1 (6.2%)	0 (0%)
Hohman et al 2014^10^	5	68.4 ± 7.5		80 ± 11.4	31 ± 25.7	122 ± 11.5	25 ± 11.9	101 ± 23	80 ± 7.1	60 ± 9.7	167 ± 9.7	3 (60%)	2 (40%)
Stephens et al 2020	9	71 ± 13.7	44.1 ± 22.7	76.1 ± 9.6								2 (22.2%)	1 (11.1%)
Celli et al 2022^7^	24	64 ± 7.9	90.6 ± 28.1	89	12.6 ± 9.4	121 ± 15.9	25 ± 15.8	96 ± 25.7	77 ± 9.1	75.4 ± 11.8	152.5 ± 18.5	12 (50%)	0 (0%)
Al-Hamdani et al 2019^3^	24	65 ± 7.1	20 ± 18.4	85 ± 13.1				110 ± 22.5			160 ± 14.0	7 (29.2%)	3 (12.5%)
Parsons et al 2005^18^	4					130	16						
Burkhart et al 2011^6^	8	75.3 ± 9.2	11 ± 4	89.8 ± 13	12.6 ± 11.1	121.9 ± 14	14.4 ± 6.2	107.5 ± 17.3	80.6 ± 9.4	78.1 ± 13.1	158.8 ± 18.1	3 (37.5%)	
Smith et Hughes 2013	21											10 (47.6%)	
Smith et al 2016	5	43.8 ± 10.6	72.0 ± 43.3	88.0 ± 19.5	13.3 ± 11.9	123.0 ± 6.7	23.0 ± 16.8	100.0 ± 19.7	75.0 ± 30.8	68.0 ± 43.8	143.0 ± 74.5	2 (40.0%)	
Ricon et al 2021^20^	5	74.0 ± 6.4	60.0 ± 32.5	82.0 ± 2.7		109.0 ± 8.9	8.0 ± 4.5	100.0 ± 7.4				2 (40.0%)	1 (20.0%)

*MEPS*, Mayo Elbow Performance Score; *DASH*, Disabilities of the Arm, Shoulder and Hand score; *F-U*, follow-up duration; *SD*, standard deviation; *PS*, prono-supination; *FE*, flexion-extension.

*NR*, not reported; values reported only when stratified by indication are explicitly available in the source.

**Table II:** Characteristics of included studies reporting results of distal humeral hemiarthroplasty performed as a salvage procedure

Author	No. of patients	Age Mean ± SD	Follow-up Mean ± SD	MEPS Mean ± SD	DASH Mean ± SD	Pronation Mean ± SD	Supination Mean ± SD	PS arc Mean ± SD	Flexion Mean ± SD	Extension loss Mean ± SD	FE arc Mean ± SD	Complications N (%)	Revisions N (%)
Heijink et al 2015^9^	5	65.4 ± 8.1	12.1 ± 12.1	75 ± 26.9	18.5 ± 22.8	85 ± 7.1	83 ± 6.7	168 ± 13	124 ± 10.8	28 ± 8.4	96 ± 18.5	3 (60%)	0 (0%)
Hohman et al 2014^10^	2	51 ± 24.5		65 ± 21.2	39 ± 32.5	100 ± 1	90 ± 7.1	180 ± 7.1	107.5 ± 24.7	22.5 ± 3.5	85 ± 21.2	0 (0%)	0 (0%)
Celli et al 2022^7^	17	61 ± 8.7	94.4 ± 32	84.4	20.3 ± 14.1	78.2 ± 7.3	78.8 ± 4.8	157 ± 12.1	117 ± 17.8	19 ± 11.9	101.4 ± 27	8 (47.1%)	1 (5.9%)
Parsons et al 2005^18^	4								121	30			
Burkhart et al 2011^6^	2	74.5 ± 4.9	16.5 ± 9.2	97.5 ± 3.5	7.1 ± 10	80 ± 14.1	85 ± 7.1	165 ± 21.2	135 ± 7.1	30 ± 0	105 ± 7.1	0 (0%)	
Smith et Hughes 2013	5											0 (0%)	
Smith et al 2016	1	46	127	85	7	90	90	180	140	20	120	0 (0%)	

*MEPS*, Mayo Elbow Performance Score; *DASH*, Disabilities of the Arm, Shoulder and Hand score; *F-U*, follow-up duration; *ACUTE*, primary surgery performed in the acute phase after fracture; *SALVAGE*, salvage surgery performed after failure of osteosynthesis; *SD*, standard deviation; *PS*, prono-supination; *FE*, flexion-extension.

### Functional outcomes

**Figure 2:**
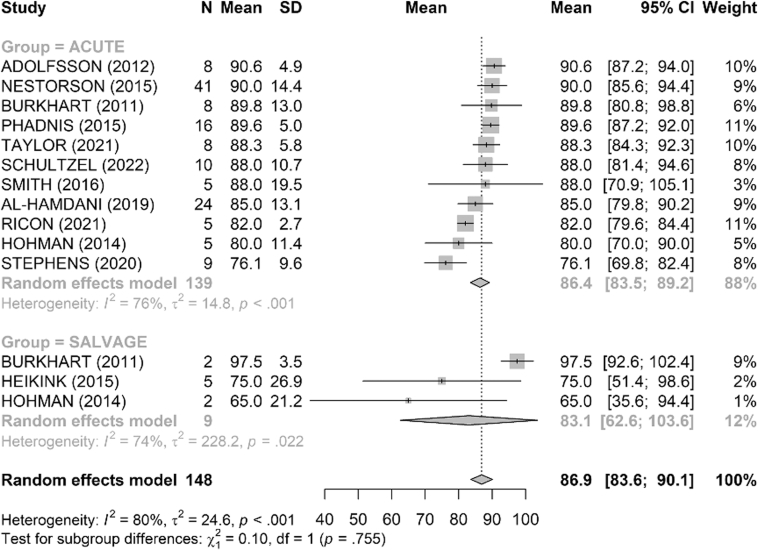
Comparison of Mayo Elbow Performance Score (MEPS, points) between acute and salvage groups. Random-effects model (inverse-variance, DerSimonian–Laird). Squares represent study estimates weighted by sample size; horizontal bars, 95% confidence intervals (CIs); diamond, pooled effect with 95% CI. Heterogeneity is reported with I^2^ and τ^2^; 95% prediction interval is provided where estimable. *SD*, standard deviation.

**Figure 3:**
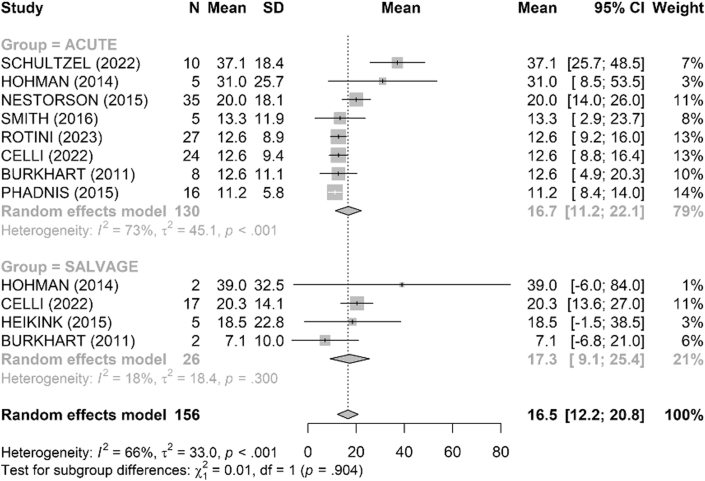
Comparison of Disabilities of the Arm, Shoulder and Hand (DASH, points) between acute and salvage groups. Random-effects model (inverse-variance, DerSimonian–Laird). Squares = study estimates; bars = 95% confidence interval (CI); diamond = pooled effect. I^2^ and τ^2^ quantify heterogeneity; 95% prediction interval shown when estimable. *SD*, standard deviation.

The MEPS functional score was reported in 15 studies (Fig. 2). The mean score was 86 points in the acute group and 83 points in the salvage group with no difference between the 2 groups (*P* = .76). The DASH score, reported in 8 studies, was similar between groups, with a mean of 16.7 in the acute group and 17.3 in the salvage group (*P* = .90) (Fig. 3).

### Mobility

**Figure 4:**
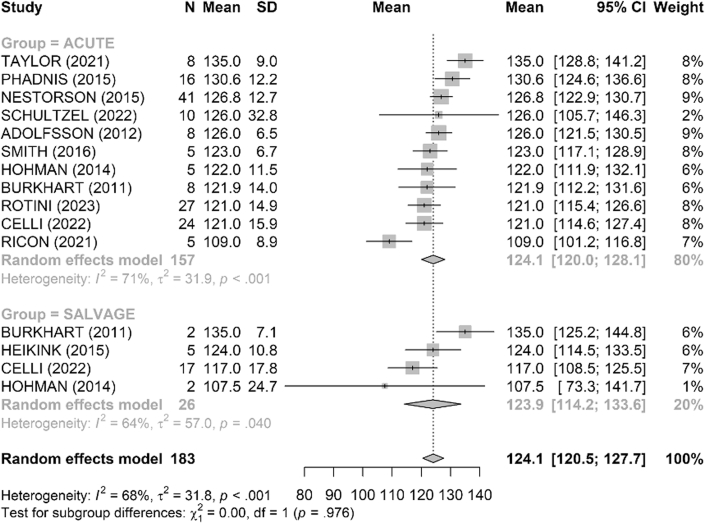
Comparison of flexion (degrees) between acute and salvage groups. Random-effects model. Squares = study estimates; bars = 95% confidence interval (CI); diamond = pooled mean difference with 95% CI. I^2^ and τ^2^ for heterogeneity; 95% prediction interval provided when estimable. *SD*, standard deviation.

**Figure 5:**
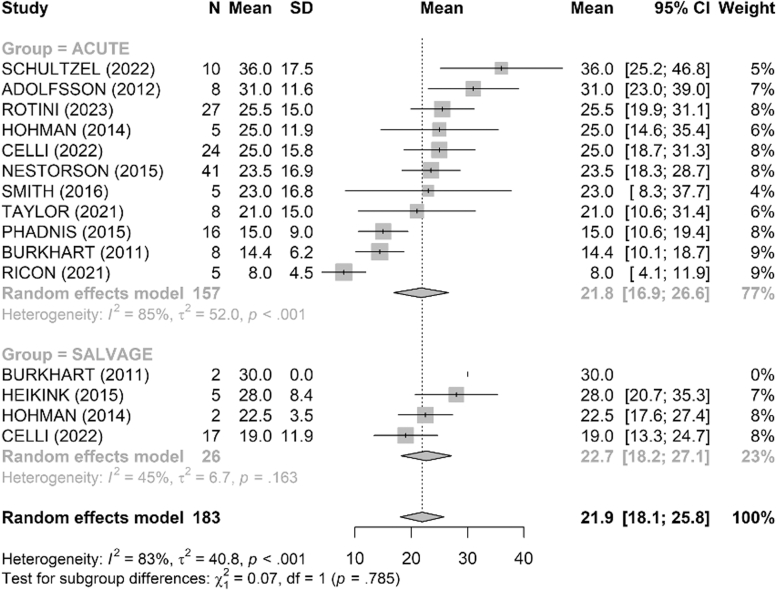
Comparison of extension loss (degrees) between acute and salvage groups. Random-effects model. Squares = study estimates; bars = 95% confidence interval (CI); diamond = pooled effect with 95% CI. I^2^ and τ^2^ reported; 95% prediction interval provided when estimable. *SD*, standard deviation.

**Figure 6:**
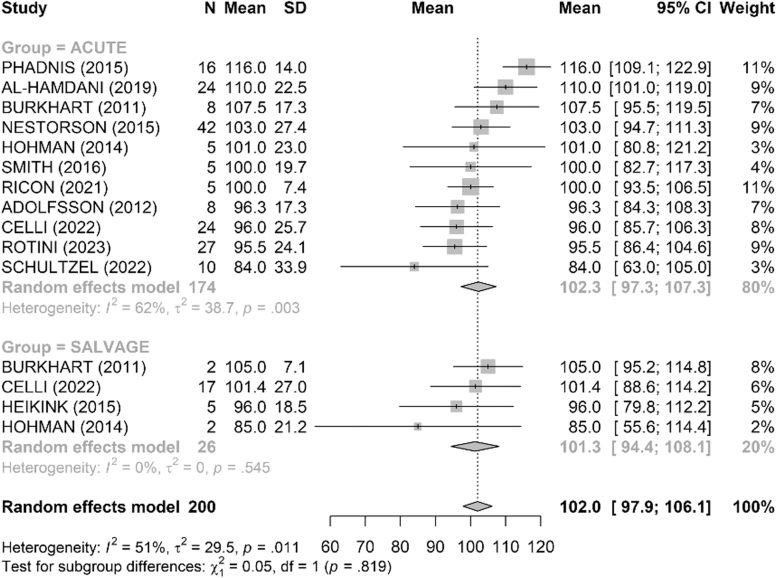
Comparison of flexion–extension arc (degrees) between acute and salvage groups. Random-effects model. Squares = study estimates; bars = 95% confidence interval (CI); diamond = pooled effect. Heterogeneity assessed with I^2^ and τ^2^; 95% prediction interval shown when estimable. *SD*, standard deviation.

**Figure 7:**
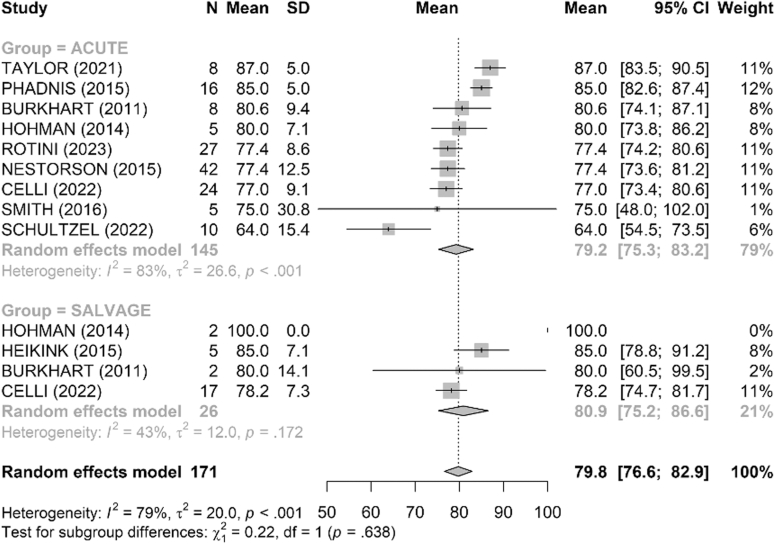
Comparison of pronation (degrees) between acute and salvage groups. Random-effects model. Squares = study estimates; bars = 95% confidence interval (CI); diamond = pooled effect with 95% CI. I^2^ and τ^2^ reported; 95% prediction interval when estimable. *SD*, standard deviation.

**Figure 8:**
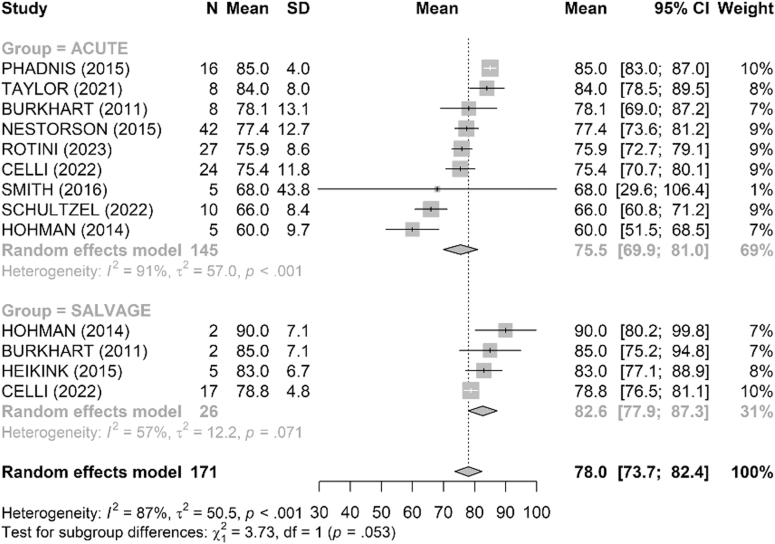
Comparison of supination (degrees) between acute and salvage groups. Random-effects model. Squares = study estimates; bars = 95% confidence interval (CI); diamond = pooled effect with 95% CI. I^2^ and τ^2^ reported; 95% prediction interval provided when estimable. *SD*, standard deviation.

**Figure 9:**
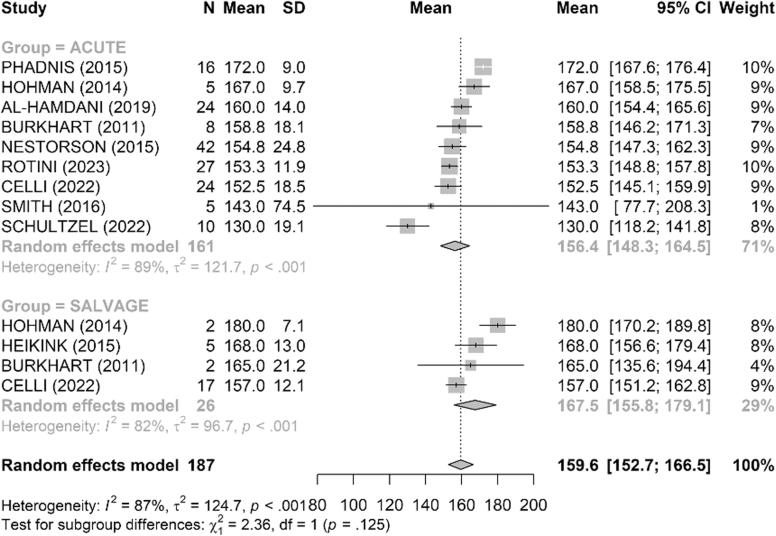
Comparison of pronation–supination arc (degrees) between acute and salvage groups. Random-effects model. Squares = study estimates; bars = 95% confidence interval (CI); diamond = pooled effect with 95% CI. Heterogeneity reported with I^2^ and τ^2^; 95% prediction interval when estimable. *SD*, standard deviation.

Range of motion was satisfactory in both groups (Figure 4, Figure 5, Figure 6, Figure 7, Figure 8, Figure 9). The mean flexion-extension arc was 102° in acute cases and 101° in salvage cases. The mean pronation-supination arc was 156° in acute cases and 157° in salvage cases. There was no significant difference between the groups for these 2 parameters. The mean loss of extension was 22° in acute cases and 23° in salvage cases, while active flexion was 124° in both groups. Pronation and supination values were also comparable, at 79° and 76° in the acute group and 81° and 83° in the salvage group, respectively.

### Complications

**Figure 10:**
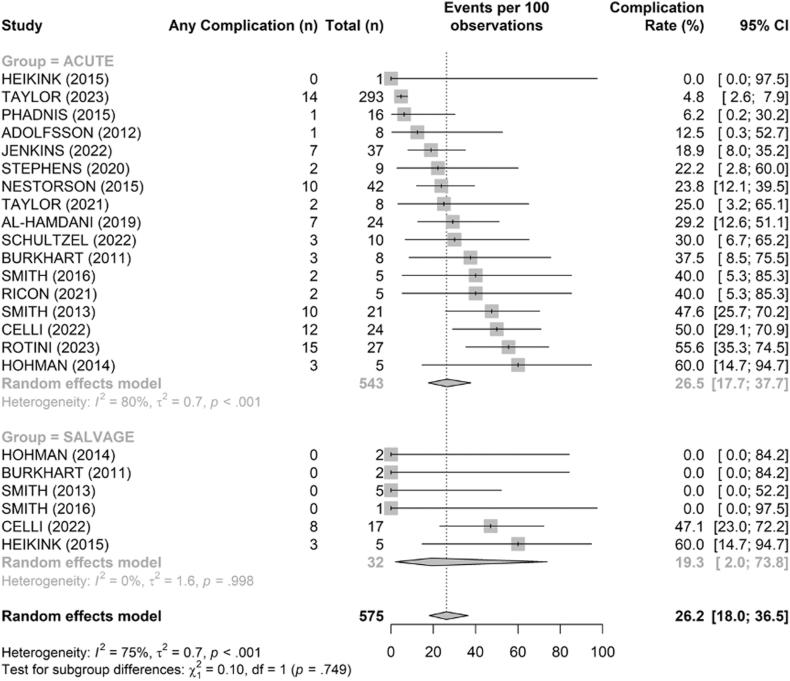
Comparison of overall complication rates (%) between acute and salvage groups. Random-effects model. Squares = study estimates; bars = 95% confidence interval (CI); diamond = pooled proportion with 95% CI. Heterogeneity reported with I^2^ and τ^2^; 95% prediction interval provided when estimable.

The pooled complication rate was 26.5% (95% confidence interval [CI] [17.7–37.7]) in acute cases and 19.3% (95% CI [2–73.8]) in salvage cases (Fig. 10). This difference was not statistically significant in our random-effects comparison (*P* = .75). Complications included cases of joint incongruity, heterotopic ossification, ulnar wear, and instability (Table III).

**Table III:** Reported crude complication rates in acute and salvage groups

	Acute	Salvage
Complications n (%)	94 (17.2%)	11 (30.6%)
Traumatic dislocation n (%)	2 (0.4%)	0 (0%)
Triceps weakness n (%)	1 (0.2%)	0 (0%)
Radial nerve palsy n (%)	1 (0.2%)	0 (0%)
Wound breakdown n (%)	6 (1.1%)	0 (0%)
Ulnar sigmoiditis n (%)	2 (0.4%)	0 (0%)
Stiffness n (%)	24 (4.4%)	4 (11.1%)
Ulnar nerve neuropathy n (%)	26 (4.8%)	4 (11.1%)
Olecranon hardwear discomfort n (%)	3 (0.5%)	0 (0%)
Infection n (%)	4 (0.7%)	0 (0%)
Fracture n (%)	5 (0.9%)	0 (0%)
Loosening n (%)	8 (1.5%)	0 (0%)
Instability n (%)	12 (2.2%)	5 (13.9%)
Humero-ulnar wear n (%)	32 (5.9%)	10 (27.8%)
Humero-radial wear n (%)	11 (2%)	5 (13.9%)
Heterotopic ossifications n (%)	66 (12.1%)	7 (19.4%)
Humero-ulnar incongruency n (%)	4 (0.7%)	2 (5.6%)
Humero-radial incongruency n (%)	6 (1.1%)	1 (2.8%)

*ACUTE*, primary surgery performed in the acute phase after fracture; *SALVAGE*, salvage surgery performed after failure of osteosynthesis.

### Revisions

**Figure 11:**
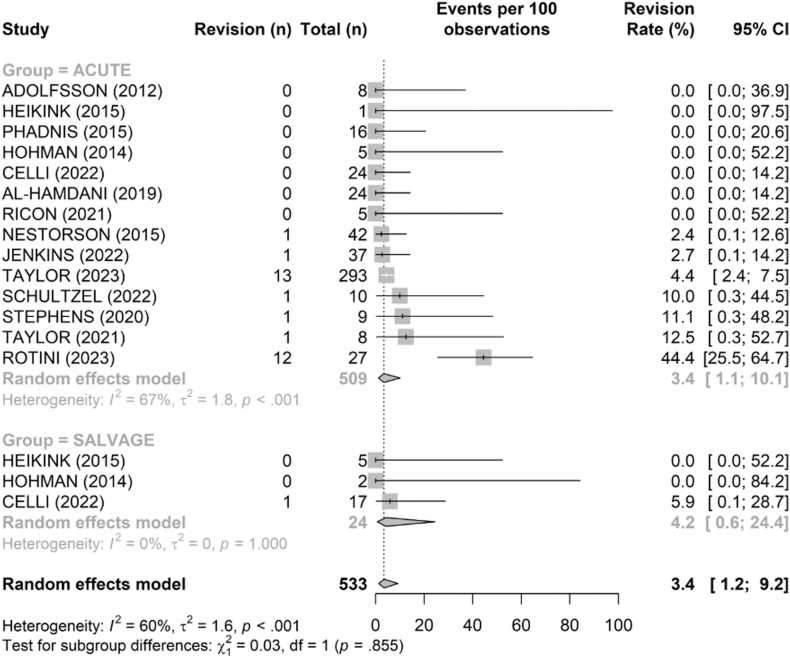
Comparison of revision rates (%) between acute and salvage groups. Random-effects model. Squares = study estimates; bars = 95% confidence interval (CI); diamond = pooled proportion with 95% CI. I^2^ and τ^2^ reported; 95% prediction interval shown when estimable.

The overall revision rate (Fig. 11) was low in both groups: 3.4% (95% CI [1.1–10.1]) in the acute group and 4.2% (95% CI [0.6–24.4]) in the salvage group, with no significant difference (*P* = .855).

## Discussion

Comminuted fractures of the distal humerus represent a significant therapeutic challenge, particularly in cases of poor bone quality or significant joint comminution. In this context, DHH is an alternative to osteosynthesis and TEA. The aim of this study was to evaluate the influence of surgical timing — acute versus salvage of failed open reduction and internal fixation (ORIF)/fracture sequelae — on functional outcomes, range of motion, and complications. Our results show that DHH performed in acute and salvage cases provided comparable functional outcomes. The pooled complication rate was also similar between the groups. These results suggest that contrary to some expectations, the salvage context did not adversely affect the functional outcomes of DHH.

The results of our meta-analysis were consistent with those reported in the literature regarding the functional efficacy of DHH for comminuted distal fractures, particularly in acute cases. Several single-center series have reported MEPS scores between 75 and 90, with satisfactory functional mobility and a moderate complication rate.^1^^,^^11^^,^^19^^,^^20^^,^^22^ Taylor et al^22^ reported a mean MEPS score of 88 for comminuted intra-articular fractures, with a mean flexion arc of 135°, pronation of 87°, and supination of 84°, results very similar to those observed in our acute group. Stephens et al^20^ studied DHHs in patients that weight bear through assistive devices preoperatively. With a mean follow-up of approximately 4 years, they reported a mean MEPS of 76 points with 11% revision, showing that this arthroplasty could be loaded with no restrictions on weight bearing/body weight patients.

In younger populations, the results were also encouraging. Smith et al^320^ reported a mean MEPS of 88 and a DASH of 12 in 6 patients under the age of 55, demonstrating that DHH can be used in a younger population. Two medium-term studies in active patients reported a low incidence of loosening and satisfactory joint ranges of motion.^9^^,^^12^

Data on salvage procedures remain limited. In the series by Parsons et al,^15^ patients operated on in nonacute settings showed slightly higher pain levels but comparable range of motion to our pooled estimates. When contextualized with the literature on TEA performed after failed ORIF, our findings are consistent with previously reported outcomes. Logli et al^10^ and Schwartz et al,^17^ although focusing on TEA, demonstrated that primary and secondary interventions yield similar functional scores and complication profiles, even after prior fixation. Reported complication rates for salvage TEA range from 20% to 35%,^10^^,^^14^^,^^16^^,^^17^ encompassing instability, polyethylene wear, and mechanical loosening—figures comparable to our pooled 19.3% complication rate for salvage DHH. These observations suggest that DHH, when technically feasible, represents a valid alternative to TEA in salvage settings, offering similar overall risks while preserving bone stock and avoiding the long-term activity restrictions associated with TEA. Taken together, both DHH and TEA series indicate that the timing of implantation—acute versus salvage—may have less influence on outcomes than patient selection and local tissue conditions.

The slightly higher pooled complication rate observed in acute cases (26.5%) compared with salvage cases (19.3%) was not statistically significant in our random-effects model (*P* = .75). This apparent difference likely reflects variability in case complexity, surgical indications, and tissue quality rather than a true disparity in risk. Importantly, no increase in revision rate was observed in the salvage group. Given the small sample size of the salvage cohort (n = 36), our analysis was underpowered to detect rare events. Larger, prospective comparative studies are warranted to confirm these findings and better define complication profiles in each setting. Interestingly, previous reports by Adolfsson et al^2^ and Seok et al^18^ suggested a slightly higher incidence of stiffness and reoperation after salvage DHH, although without major impact on functional outcomes or patient satisfaction — results not corroborated by our pooled analysis.

This meta-analysis directly compared DHH in acute versus salvage situations, which was not the case in most previous series, often limited to a single surgical indication.

Finally, some authors have suggested that DHH should be preferred to TEA due to a lower rate of mechanical complications. Celli et al^5^ and Burkhart et al^4^ reported good bone integration, a moderate risk of ulnar wear, and a low need for conversion. These findings support further exploration of the role of DHH in the therapeutic arsenal.

Several hypotheses could explain why the functional results were similar between acute and salvage surgery: the DHH is a nonconstrained implant that adapts to the anatomy and resolves intra-articular problems in both acute and chronic cases. Patients undergoing salvage surgery may have benefited from a period of prior rehabilitation and/or better surgical selection. It is also possible that patients undergoing salvage surgery have lower functional expectations. Finally, recent advances in implants and surgical techniques (e.g. Latitude EV, ligament fixation techniques described by Taylor^22^) have probably improved outcomes in acute and salvage cases. Further improvement of techniques and implants is very important to further improve clinical and radiological outcomes. From a clinical point of view, these results support the idea that the salvage context alone should not be a contraindication for performing a DHH. However, it remains essential to assess the condition of the soft tissues, bone stock, extensor mechanism, ligaments, and potential for functional rehabilitation.

This meta-analysis has several strengths: a large number of studies included (n = 18), a rigorous synthesis according to PRISMA recommendations, and the analysis of functional criteria objectified by validated scores. It covers a wide spectrum of patients and surgical contexts. However, it also has limitations. Most of the included studies are retrospective, with a low to moderate level of evidence. The number of patients in the ‘salvage’ group remains relatively small (n = 36), which limits statistical power. The heterogeneity of surgical techniques, implants used, and follow-up also complicates comparisons. Finally, some criteria such as subjective satisfaction or return to work were not always reported.

Further research on this topic is needed in the form of prospective comparative studies or randomized controlled trials. Studies evaluating quality of life, the cost-effectiveness of DHH versus TEA, or long-term radiographic outcomes (ulnar wear, osteolysis, stability) would be particularly relevant. A prospective multicenter database, including standardized criteria (MEPS score, DASH, satisfaction, revisions), would be a valuable tool for better defining the optimal indications.

Our study shows that DHH offered satisfactory functional results and acceptable complication rates, whether performed acutely or as a salvage procedure after failed osteosynthesis/fracture sequelae. These data support the validity of this surgical option in both contexts and require for a more systematic evaluation of the role of DHH in the decision-making algorithm for complex elbow fractures.

## Conclusion

DHH provides comparable functional outcomes and complication profiles whether performed acutely for unreconstructable fractures or as a salvage procedure after failed ORIF. These findings suggest that surgical timing alone should not be considered a determinant of prognosis. When patient selection and surgical technique are appropriate, DHH remains a reliable reconstructive option in both contexts. Further prospective, adequately powered comparative studies are warranted to refine indications and better define long-term outcomes.

## Disclaimers:

Funding: The authors received no financial support for the research, authorship, and/or publication of this article.

Conflicts of interest: The authors, their immediate families, and any research foundations with which they are affiliated have not received any financial payments or other benefits from any commercial entity related to the subject of this article.

Given his role as Editor in Chief, Pierre Mansat had no involvement in the peer-review of this article and has no access to information regarding its peer-review. Full responsibility for the editorial process for this article was delegated to William Mallon.

## References

[1] Adolfsson L., Hammer R. (2006). Elbow hemiarthroplasty for acute reconstruction of intraarticular distal humerus fractures: a preliminary report involving 4 patients. Acta Orthop.

[2] Adolfsson L., Nestorson J. (2012). The Kudo humeral component as primary hemiarthroplasty in distal humeral fractures. J Shoulder Elbow Surg.

[3] Borenstein M., Hedges L.V., Higgins J.P.T., Rothstein H.R. (2010). A basic introduction to fixed-effect and random-effects models for meta-analysis. Res Synth Method.

[4] Burkhart K.J., Nijs S., Mattyasovszky S.G., Wouters R., Gruszka D., Nowak T.E. (2011). Distal humerus hemiarthroplasty of the elbow for comminuted distal humeral fractures in the elderly patient. J Trauma Inj Infect Crit Care.

[5] Celli A., Ricciarelli M., Guerra E., Bonucci P., Ritali A., Cavallo M. (2022). Elbow hemiarthroplasty for acute distal humeral fractures and their sequelae: medium- and long-term follow-up of 41 cases. J Shoulder Elbow Surg.

[6] Charissoux J.-L., Mabit C., Fourastier J., Beccari R., Emily S., Cappelli M. (2008). Comminuted intra-articular fractures of the distal humerus in elderly patients. Rev Chir Orthop Reparatrice Appar Mot.

[7] Githens M., Yao J., Sox A.H.S., Bishop J. (2014). Open reduction and internal fixation versus total elbow arthroplasty for the treatment of geriatric distal humerus fractures: a systematic review and meta-analysis. J Orthop Trauma.

[8] Jonsson E.Ö., Ekholm C., Hallgren H.B., Nestorson J., Etzner M., Adolfsson L. (2024). Elbow hemiarthroplasty and total elbow arthroplasty provided a similar functional outcome for unreconstructable distal humeral fractures in patients aged 60 years or older: a multicenter randomized controlled trial. J Shoulder Elbow Surg.

[9] Jukes C.P., Dirckx M., Phadnis J. (2021). Current concepts in distal humeral hemiarthroplasty. J Clin Orthop Trauma.

[10] Logli A.L., Shannon S.F., Boe C.C., Morrey M.E., O’Driscoll S.W., Sanchez-Sotelo J. (2020). Total elbow arthroplasty for distal humerus fractures provided similar outcomes when performed as a primary procedure or after failed internal fixation. J Orthop Trauma.

[11] Luciani A.M., Baylor J., Akoon A., Grandizio L.C. (2023). Controversies in the management of Bicolumnar fractures of the distal humerus. J Hand Surg.

[12] McNeil D.S., Barton K.I., Faber K.J. (2023). A case report: instability after distal humerus hemiarthroplasty leading to revision with a total elbow arthroplasty. JSES Rev Rep Tech.

[13] Page M.J., McKenzie J.E., Bossuyt P.M., Boutron I., Hoffmann T.C., Mulrow C.D. (2021). The PRISMA 2020 statement: an updated guideline for reporting systematic reviews. Plos Med.

[14] Palladino S., Baldairon F., Godet J., Clavert P. (2024). Outcomes of total elbow arthroplasty in the treatment of distal humeral fractures in the elderly: a retrospective cohort comparison between primary arthroplasty and arthroplasty secondary to failed internal fixation. J Shoulder Elbow Surg.

[15] Parsons M., O’Brien R.J., Hughes J.S. (2005). Elbow hemiarthroplasty for acute and salvage reconstruction of intra-articular distal humerus fractures. Tech Shoulder Elbow Surg.

[16] Prasad N., Dent C. (2008). Outcome of total elbow replacement for distal humeral fractures in the elderly: a comparison of primary surgery and surgery after failed internal fixation or conservative treatment. J Bone Joint Surg Br.

[17] Schwartz J.M., Ramamurti P., Werner B.C., Dacus A.R. (2024). Does timing of total elbow arthroplasty after distal humerus fracture affect 2-year complication rates?. J Shoulder Elbow Surg.

[18] Seok H.-G., Park J.-J., Park S.-G. (2022). Comparison of the complications, reoperations, and clinical outcomes between open reduction and internal fixation and total elbow arthroplasty for distal humeral fractures in the elderly: a systematic review and meta-analysis. J Clin Med.

[19] Smith G.C.S., Bayne G., Page R., Hughes J.S. (2016). The clinical outcome and activity levels of patients under 55 years treated with distal humeral hemiarthroplasty for distal humeral fractures: minimum 2-year follow-up. Shoulder Elbow.

[20] Stephens J., Kohrs B., Bushnell L., Gabriel S., Brent Bamberger H. (2020). Distal humerus fractures managed with elbow Hemiarthroplasty. J Shoulder Elbow Arthroplasty.

[21] Sterne J.A., Hernán M.A., Reeves B.C., Savović J., Berkman N.D., Viswanathan M. (2016). ROBINS-I: a tool for assessing risk of bias in non-randomised studies of interventions. BMJ.

[22] Taylor J.R., Shea K.E., Clark C.F., Kelly J.D., Schrumpf M.A. (2021). Elbow hemiarthroplasty for intra-articular distal humerus fractures: results and technique. JSES Rev Rep Tech.

[23] Vauclair F., Goetti P., Nguyen N.T.V., Sanchez-Sotelo J. (2020). Distal humerus nonunion: evaluation and management. EFORT Open Rev.

[24] Viechtbauer W. (2010). Conducting meta-analyses in *R* with the metafor package. J Stat Soft.

